# Dihydrochalcone Derivative Induces Breast Cancer Cell Apoptosis via Intrinsic, Extrinsic, and ER Stress Pathways but Abolishes EGFR/MAPK Pathway

**DOI:** 10.1155/2019/7298539

**Published:** 2019-10-22

**Authors:** Wasitta Rachakhom, Patompong Khaw-on, Wilart Pompimon, Ratana Banjerdpongchai

**Affiliations:** ^1^Department of Biochemistry, Faculty of Medicine, Chiang Mai University, Chiang Mai 50200, Thailand; ^2^Laboratory of Natural Products, Faculty of Science, Lampang Rajabhat University, Lampang 52100, Thailand

## Abstract

Dihydrochalcone derivatives are active compounds that have been purified from the Thai medicinal plant *Cyathostemma argenteum*. The objectives of this study were to investigate the effects of two dihydrochalcone derivatives on human breast cancer MDA-MB-231 and MCF-7 cell proliferation and to study the relevant mechanisms involved. The two dihydrochalcone derivatives are 4′,6′-dihydroxy-2′,4-dimethoxy-5′-(2″-hydroxybenzyl)dihydrochalcone (compound 1) and calomelanone (2′,6′-dihydroxy-4,4′-dimethoxydihydrochalcone, compound 2), both of which induced cytotoxicity toward both cell lines in a dose-dependent manner by using MTT assay. Treatment with both derivatives induced apoptosis as determined by annexin V-FITC/propidium iodide employing flow cytometry. The reduction of mitochondrial transmembrane potential (staining with 3,3′-dihexyloxacarbocyanine iodide, DiOC_6_, employing a flow cytometer) was established in the compound 1-treated cells. Compound 1 induced caspase-3, caspase-8, and caspase-9 activities in both cell lines, as has been determined by specific colorimetric substrates and a spectrophotometric microplate reader which indicated the involvement of both the extrinsic and intrinsic pathways. Calcium ion levels in mitochondrial and cytosolic compartments increased in compound 1-treated cells as detected by Rhod-2AM and Fluo-3AM intensity, respectively, indicating the involvement of the endoplasmic reticulum (ER) stress pathway. Compound 1 induced cell cycle arrest via enhanced *atm* and *atr* expressions and by upregulating proapoptotic proteins, namely, Bim, Bad, and tBid. Moreover, compound 1 significantly inhibited the EGFR/MAPK signaling pathway. In conclusion, compound 1 induced MDA-MB-231 and MCF-7 cell apoptosis via intrinsic, extrinsic, and ER stress pathways, whereas it ameliorated the EGFR/MAPK pathway in the MCF-7 cell line. Consequently, it is believed that compound 1 could be effectively developed for cancer treatments.

## 1. Introduction

Cancer incidence is rapidly growing worldwide, and breast cancer is the most common cause of cancer-related deaths among women. There were about 2.1 million newly diagnosed female breast cancer cases in 2018 [1]. In various treatments, chemotherapeutic drugs, surgery, radiation, and immune therapies induced breast cancer cells to undergo apoptosis. However, cancer cells may escape and resist cell death against the treatments and consequently become even more aggressive as cancer cells. It has been proven that chemotherapy and hormonal therapy improve the prognosis of postoperation breast cancer patients [2]. Recently, the most effective treatment for breast cancer has been based on an abnormal oncogene of the cancer cells, while the expressions of the estrogen receptor (ER) and progesterone receptor (PR) are used as prognostic factors. Notably, ER-positive (ER^+^) tumors are more responsive to hormonal therapies than ER-negative (ER^−^) tumors. Additionally, MCF-7 and MDA-MB-231 cells are considered HER2-negative breast cancer cells. The difference between MCF-7 and MDA-MB-231 cells is that MCF-7 cells possess the capability of expression of an estrogen receptor (ER^+^), but not in MDA-MB-231 cells (ER^−^). Hence, the MDA-MB-231 cell is also progesterone-negative, allowing it to be more aggressive than MCF-7 since it is a triple-negative breast cancer cell [3, 4].

Chemotherapeutic drugs are the main forms of treatment for cancer, but the responses are limited due to the drugs' toxicity toward normal cells and their anticancer resistance [5]. The chemodrugs are used to inhibit metabolic pathways that are crucial for cell division. Side effects of chemotherapeutic drugs are an important consideration for cancer patients, and diminishing the cytotoxicity of drugs to normal cells is the most important aspect for cancer research [6]. Inhibition of the proliferation and induction of cancer cell death via apoptosis are the main targets for cancer treatments [7]. Mechanisms of apoptosis play important roles in the pathogenesis of many diseases. Hence, apoptosis is significant in terms of both carcinogenesis and cancer treatments [8]. Apoptosis is the main type of cell death that is induced by most of the frontline chemotherapeutic agents [9]. In the process of apoptotic cell death, the morphology of the apoptosis cells alters as by way of cell membrane blebbing, cell shrinkage, nuclear condensation, and chromatin fragmentation [10]. Apoptosis is a regulated and efficient form of programmed cell death that involves multiple factors. Caspases play a major and central role in apoptotic mechanisms. There are three pathways to activate caspases. First is the intrinsic or mitochondrial pathway; second is the extrinsic or death receptor pathway [11]; and finally, there is the endoplasmic reticulum (ER) stress pathway [8].

Mitogen-activated protein kinases (MAPKs) are serine/threonine kinases, which phosphorylate their specific substrates as serine and/or threonine residues to regulate gene expression, mitosis, proliferation, motility, metabolism, and programmed apoptotic cell death [12]. MAPKs possess three subfamilies including extracellular signal-regulated kinases (ERKs), c-Jun N-terminal kinases (JNKs), and p38-MAPKs. It has been reported that ERKs are important for cell survival, whereas JNKs and p38-MAPKs are involved in apoptosis [13]. It has been reported that p38 gamma promotes triple-negative breast cancer (TNBC) development and progression by stimulating cancer stem-like cell expansion (CSC) [14]. Human breast cancer MCF-7 and MDA-MB-231 cell progressions have been promoted by p38 delta MAPK [15]. Therefore, inhibition of p38-MAPK activity may be a novel strategy for the treatment of breast cancer.

The pathway that regulates cell survival/death involves the epidermal growth factor receptor (EGFR) pathway. EGFR, a receptor tyrosine kinase (RTK), is activated to promote cell proliferation, motility, and survival via various downstream signaling pathways [16]. Inhibitors of EGFR agents have been used in clinical trials, such as gefitinib, neratinib, and erlotinib. These affect breast cancer cell proliferation and have been used for cancer treatments [17]. Recently, it was found that verrucarin A induces ROS levels in breast cancer MDA-MB-231 cells resulting in the activation of p38-MAPK and the inhibition of EGFR/Akt/ERK signaling cascade to cause cell deaths [18]. EGFR signaling promotes tumor growth and immune escape via induction of the glycolysis pathway in triple-negative breast cancer MDA-MB-231 cells [19]. Thus, EGFR activation can be a target for breast cancer treatment as well as MAPK inhibition.

As a consequence of cancer resistance and the adverse side effects of target-oriented therapies, novel strategies for cancer treatment are needed [20]. The mechanism of natural product-induced cancer cell death via apoptosis has been studied for more than three decades [21]. Sesquiterpenes, flavonoids, alkaloids, diterpenoids, and polyphenols represent large and diverse groups of natural compounds that are found in fruits, vegetables, and medicinal plants possessing anticancer properties [22].

Both dihydrochalcones were purified compounds obtained from *Cyathostemma argenteum*. *Cyathostemma* species comprised of six species including *longipes, micranthum, siamensis, viridiflorum*, *wrayi*, and *Cyathostemma argenteum*, which are all widely grown in Thailand [23]. In Malaysian and Thai traditional medicine, *C. argenteum* has been used as an antispasmodic agent to alleviate pain associated with menstruation and following child-birth [24]. Previous investigations of the *Cyathostemma* genus revealed that the methanolic extract from its root and stem bark is effective against breast cancer [23, 25]. Compound 1 has been reported to inhibit TNF-*α*-induced NF-κB activation [26]. Dihydrochalcone derivatives, 4′,6′-dihydroxy-2′,4-dimethoxy-5′-(2″-hydroxybenzyl)dihydrochalcone (compound 1) and 4′,6′-dihydroxy-2′,4-dimethoxydihydrochalcone (compound 2), isolated from the leaves and twigs of *Cyathostemma argenteum*, possess anti-inflammatory activity [23]. There have not yet been any reports on the anticancer activity of these dihydrochalcone derivatives: compound 1 and compound 2; therefore, the aims of the present study were to investigate the inhibitory effects of the active compounds obtained from *C. argenteum* on cancer cell proliferation. The mode and mechanisms of cell death induced by the compounds were demonstrated by using human breast MDA-MB-231 and MCF-7 cancer cell lines as an *in vitro* model.

## 2. Materials and Methods

### 2.1. Chemicals

**Figure 1:**
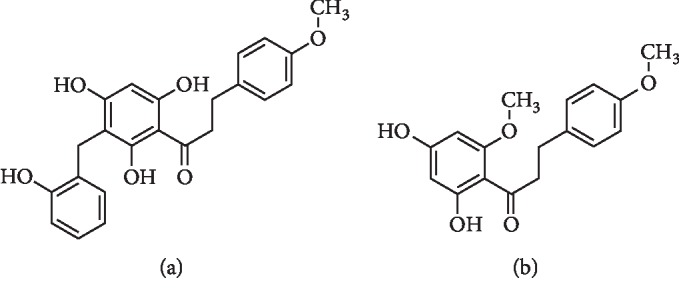
Chemical structures of dihydrochalcone derivatives used in the study. Compound 1 (4′,6′-dihydroxy-2′,4-dimethoxy-5'-(2″-hydroxybenzyl)dihydrochalcone) (a) and compound 2 (calomelanone; 2′,6′-dihydroxy-4,4′-dimethoxydihydrochalcone) (b).

A dihydrochalcone derivative, 4′,6′-dihydroxy-2′,4-dimethoxy-5′-(2″-hydroxybenzyl)dihydrochalcone, compound 1 (Figure 1(a)), is a purified compound extracted from the leaves and twigs of *Cyathostemma argenteum.* It was provided as a purified compound by Associate Professor Wilart Pompimon. The leaves and twigs of *C. argenteum* (*Annonaceae*) were collected in October 2011 from a swamp forest in Ubon Ratchathani Province, Thailand. The herb was identified by Mr. Narong Nantasean, from the Forest Herbarium, Department of National Parks, Wildlife and Plant Conservation, Ministry of National Resources and Environment, Bangkok, Thailand. A voucher specimen (BKF.18053) was deposited in the herbarium of this institute [23]. The other dihydrochalcone derivative also isolated from this plant was calomelanone, 2′,6′-dihydroxy-4,4′-dimethoxydihydrochalcone (compound 2, Figure 1(b)). Calomelanone (product code: FD65868) was purchased from Carbosynth (Carbosynth Ltd., Berkshire, UK).

Dulbecco's modified Eagle's medium (DMEM), fetal bovine serum, streptomycin, and penicillin G sodium were obtained from Gibco BRL (Thermo Fisher Scientific Inc., Waltham, MA, USA). Dimethyl sulfoxide (DMSO), 3-(4,5-dimethyl)-2,5-diphenyl tetrazolium bromide (MTT), propidium iodide (PI), Ficoll–Hypaque reagent (HISTOPAQUE®-1077), and 3,3′-dihexyloxacarbocyanine iodide (DiOC_6_) were obtained from Sigma-Aldrich (St. Louis, MO, USA). Annexin V-FITC-FLUOS kit and protease inhibitor cocktail were obtained from Roche (Indianapolis, IN, USA). The substrates of caspase-9 LEHD-para-nitroaniline (LEHD-*p*-NA), caspase-8 (IETD-*p*-NA), caspase-3 (DEVD-*p*-NA), and RPMI-1640 medium were obtained from Invitrogen (Thermo Fisher Scientific Inc., Waltham, MA, USA).

### 2.2. Cell Culture

Human invasive breast cancer MDA-MB-231 cells, human noninvasive breast cancer MCF-7 cells, and murine fibroblast NIH3T3 cells were obtained from Professor Prachya Kongtawelert at the Excellence Center of Tissue Engineering and Stem Cells, Department of Biochemistry, Faculty of Medicine, Chiang Mai University. Cells were cultured in DMEM, supplemented with 10% fetal bovine serum, 25 mM sodium bicarbonate (NaHCO_3_), 20 mM HEPES, 100 units/ml penicillin G, and 100 *μ*g/ml streptomycin at 37°C and 5% CO_2_. Peripheral blood mononuclear cells (PBMCs) were isolated from heparinized blood obtained from adult volunteers and from blood-donating volunteers at the Blood Bank Unit, Maharaj Nakorn Chiang Mai Hospital which was affiliated with the Faculty of Medicine, Chiang Mai University, Chiang Mai, Thailand. Each blood donor was informed of the objectives and each one signed the written consent form in compliance with the terms for each individual volunteer according to the Institutional Review Board, Research Ethics Committee, Faculty of Medicine, Chiang Mai University. PBMCs were isolated by density gradient centrifugation using Ficoll–Hypaque reagent according to standard protocols. Cells were cultured in RPMI-1640 medium supplemented with 10% heat-inactivated fetal bovine serum, 2 mM glutamine, 100 units/ml penicillin G, and 100 *μ*g/ml streptomycin at 37°C and 5% CO_2_. PBMCs (3 × 10^6^ cells/ml) were treated with compounds 1 and 2 at the indicated concentrations and for indicated durations [27].

### 2.3. Cell Cytotoxic Assay

MDA-MB-231 and MCF-7 cells (5 × 10^4^ cells/ml), normal PBCMs (3 × 10^6^ cells/ml), and murine fibroblast NIH3T3 cells (5 × 10^4^ cells/ml) were treated with compounds 1 and 2 at various concentrations for various durations. Cell viability was determined by using MTT assay and then comparing the results with untreated cells. Briefly, MTT dye was added to the cell suspension at the final concentration of 100 *μ*g/ml and it was then incubated for 4 hours at 37°C in a humidified 5% CO_2_ atmosphere. The medium was removed, and the violet crystal was dissolved with dimethyl sulfoxide (DMSO). The absorbance was measured at 540 nm and a reference wavelength at 630 nm using a microplate reader (BioTek, Winooski, VT, USA). The percentage of cell viability was calculated, and 10%, 20%, and 50% inhibitory concentrations (IC_10_, IC_20_, and IC_50_) were determined to compare the sensitivity of the compounds or drugs. These concentrations were applied in further experiments [28].

### 2.4. Apoptosis Assay

MDA-MB-231 and MCF-7 cells were treated with compounds 1 and 2 at various doses as indicated. The cells were washed twice with PBS and then stained with annexin V-fluorescein isothiocyanate (FITC) and propidium iodide (PI) for 15 minutes. The stained cells were measured using a flow cytometer and then analyzed with CellQuest (a software program) (Becton Dickinson, Franklin Lakes, NJ, USA) [29].

### 2.5. Fluorescence Microscopy

Various doses of compound 1 were selected to treat MDA-MB-231 and MCF-7 cells in the following experiments. The cells were cultured on culture slides and treated with compound 1 for 24 hours. After that, the slides were fixed in cold absolute methanol and stained with 10 *μ*g/ml propidium iodide (PI). Apoptotic morphology was determined under a fluorescence microscope. Apoptosis positive cells were counted from a total of 200 cells obtained from three independent experiments [30].

### 2.6. Measurement for Mitochondrial Transmembrane Potential (MTP) Disruption

MDA-MB-231 and MCF-7 cells were treated with compound 1 for 24 hours. The cells were then incubated with 3,3′ dihexyloxacarbocyanine iodide (DiOC_6_) at 40 nM (final concentration) for 15 minutes at 37°C, and the loss of mitochondrial transmembrane potential (MTP) was determined using a flow cytometer (Becton Dickinson, Franklin Lakes, NJ, USA) [31].

### 2.7. Determination of Cytosolic and Mitochondrial Calcium Ion Levels

The cytosolic Ca^2+^ level was determined by using 10 *μ*M fluorescence dye at a final concentration of Fluo-3AM in the FITC setting, and the mitochondrial Ca^2+^ level was determined by employing 250 nM of fluorescence dye at a final concentration of Rhod-2AM in the PE setting. After MDA-MB-231 and MCF-7 cells were treated with compound 1 for 24 hours, the cells were incubated with each fluorescence dye for 15 minutes at 37°C, washed twice with PBS, and analyzed by flow cytometry. In each analysis, 10,000 events were recorded and analyzed by the CellQuest program (Becton Dickinson, Franklin Lakes, NJ, USA) [30].

### 2.8. Determination of Caspase-3, Caspase-8, and Caspase-9 Activities

MDA-MB-231 and MCF-7 cells were incubated with compound 1 at various doses for 24 hours. The treated cells were harvested and washed twice with ice-cold PBS. The cell pellets were lysed with lysis buffer for 30 minutes on ice. The chromogenic substrate of each type of caspase, viz., caspase-3, Asp-Glu-Val-Asp-*p*-NA (DEVD-*p*-NA); caspase-8, Ile-Glu-Thr-Asp-*p*-NA (IETD-*p*-NA); and caspase-9, Leu-Glu-His-Asp-*p*-NA (LEHD-*p*-NA), was added to the reaction buffer of the cell lysate. The cell lysate was incubated with each substrate for 60 minutes, and then caspase-3, caspase-8, and caspase-9 activities were measured using a microplate reader at 405 nm (BioTek, Winooski, VT, USA) [32].

### 2.9. Immunoblotting

The proteins investigated in this study included Bim, Bad, Bid, tBid, and *β*-actin. Antibodies of these proteins were purchased from Abcam, UK. Immunoblotting was performed as previously reported [29]. Briefly, after compound 1 treatment, the cells were lysed with RIPA buffer containing a protease inhibitor. Protein concentrations were determined by Bradford assay kit. The cell lysate was loaded onto 15% SDS-PAGE and transferred to the nitrocellulose membrane. The membrane was blocked with 5% nonfat milk in PBS containing 0.2% Tween-20. The membrane was then incubated with rabbit polyclonal antibodies to Bad (phospho-S136, ab28824), Bim (ab15184), and Bid and tBid (ab129192), followed by appropriate horseradish peroxidase (HRP)-conjugated secondary antibodies (1 : 20,000). Protein bands were detected on X-ray film with Super Signal West Pico Chemiluminescent Substrate. The bands were analyzed by a densitometer and compared to the control protein actin.

### 2.10. Determination of Gene Expression by Real-Time Reverse Transcription-Polymerase Chain Reaction (Real-Time RT-PCR)

After compound 1 treatment, the cells were collected and RNA was isolated from the cell pellets by using the Illustra RNAspin Mini Kit (GE Healthcare, UK). Total RNA was reversed to complementary DNA (cDNA) using Tetro cDNA Synthesis Kit (Bioline Reagents Ltd., USA). Quantitative real-time PCR assays were performed by the SensiFAST™ SYBR®Lo-ROX Kit (Bioline Reagents Ltd., USA), and the reaction solution was run by the QuantStudio™ 6 Flex Real-Time PCR System (Thermo Fisher Scientific Inc., USA). The data were analyzed by using QuantStudio™ Real-Time PCR software. All the data were normalized by using the *GAPDH* gene. The details of all gene primers are listed in Table 1.

### 2.11. Determination of EGFR/MAPK Pathway by FlowCellect™ EGFR/MAPK Activation Detection Kit

After the cells were treated with compound 1 for 4 hours, the cells were collected and then a Millipore FlowCellect™ EGFR/MAPK kit was used as has been described in the protocol. EGFR/MAPK activation was detected using an EGFR/MAPK Activation Detection kit that included two directly conjugated phospho-specific antibodies: pEGFR(Tyr1173)-Alexa Fluor 488 and pERK (Thr202/Tyr204, Thr185/Tyr187)-PE. Each sample was analyzed by (Guava®) flow cytometry analysis.

### 2.12. Drug Combination Assay

MDA-MB-231 or MCF-7 cells were cotreated with various concentrations of compound 1 and conventional therapeutic drugs at a minimum-resistance dose for 24 hours. The chemotherapeutic drugs used were imatinib, sorafenib, Smac mimetic (BV6), and doxorubicin. After combined treatments, cell viability was determined by using MTT assay and a function of the effect level (Fa) values was calculated. The CompuSyn Software was applied for determination of the combination index (CI) value. The efficiency of the combined drugs with compound 1 was investigated by the modified method of Khaw-on [30].

### 2.13. Statistical Analysis

All data are expressed as mean ± standard deviation of the mean (SD).

The biochemical data were assessed with one-way analysis of variance (ANOVA) (Kruskal–Wallis analysis) at the limits of ^*∗*^*p* < 0.05, ^*∗∗*^*p* < 0.01, ^*∗∗∗*^*p* < 0.001, and ^*∗∗∗∗*^*p* < 0.0001 from three independent experiments that were conducted in triplicate to indicate statistical significance. All tests were calculated with the commercially available software GraphPad Prism (GraphPad Software, San Diego, CA, USA). Comparison of two variables was performed by using the Mann–Whitney *U* test.

## 3. Results

### 3.1. Cytotoxic Effect of Compound 1 and Compound 2 on Human Breast Cancer MDA-MB-231 and MCF-7 Cells

**Figure 2:**
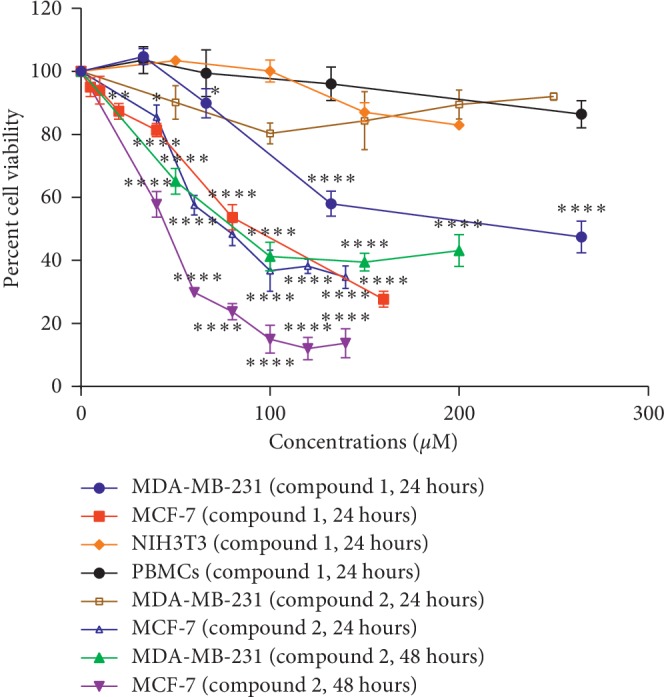
Cell cytotoxicity of dihydrochalcone derivatives against various cancer cell lines compared to normal cells. Various cell types, both normal and cancer cells, were investigated for the cytotoxicity of compound 1 and compound 2 toward MDA-MB-231, MCF-7, NIH3T3 cells, and PBMCs after incubation for 24 and 48 hours by MTT assay. ^*∗*^*p* < 0.05 and ^*∗∗∗∗*^*p* < 0.0001.

After treating cells with compound 1 and compound 2, compound 1 was found to be toxic to both MDA-MB-231 and MCF-7 after 24 hours of incubation, but was less toxic to NIH3T3 and PBMCs (with IC_50_ values of more than 200 *μ*M for both latter cell types). The inhibitory concentrations at 24 h or IC_10_, IC_20_, and IC_50_ of compound 1 on MDA-MB-231 were 57.1 ± 3.5, 84.8 ± 2.8, and 232.7 ± 3.9 *μ*M, and on MCF-7 were 22.8 ± 3.7, 45.7 ± 4.8, and 88.3 ± 5.4 *μ*M, respectively. Compound 2 was also found to be toxic to MDA-MB-231 and MCF-7 at 48 hours, but was not toxic against MDA-MB-231 at 24 hours. Compound 2 was incubated longer than compound 1 to obtain the IC_50_ at the same level of toxicity in both cancer cells which was two digits of IC_50_. At 48 hours of treatment, IC_50_ of compound 2 on MDA-MB-231 was 85.9 ± 5.3 *μ*M, and for MCF-7, it was 46.2 ± 4.7 *μ*M, respectively. Hence, compound 1 was more toxic to both cells than compound 2 in terms of a sensitivity to cancer when compared to normal cells as follows: MCF-7 > MDA-MB-231 > NIH3T3 > PBMC cells as determined by IC_50_ levels of both compounds (Figure 2 and Tables 2 and 3).

### 3.2. Compound 1 and Compound 2 Induced Apoptosis in MDA-MB-231 and MCF-7 Cells

**Figure 3:**
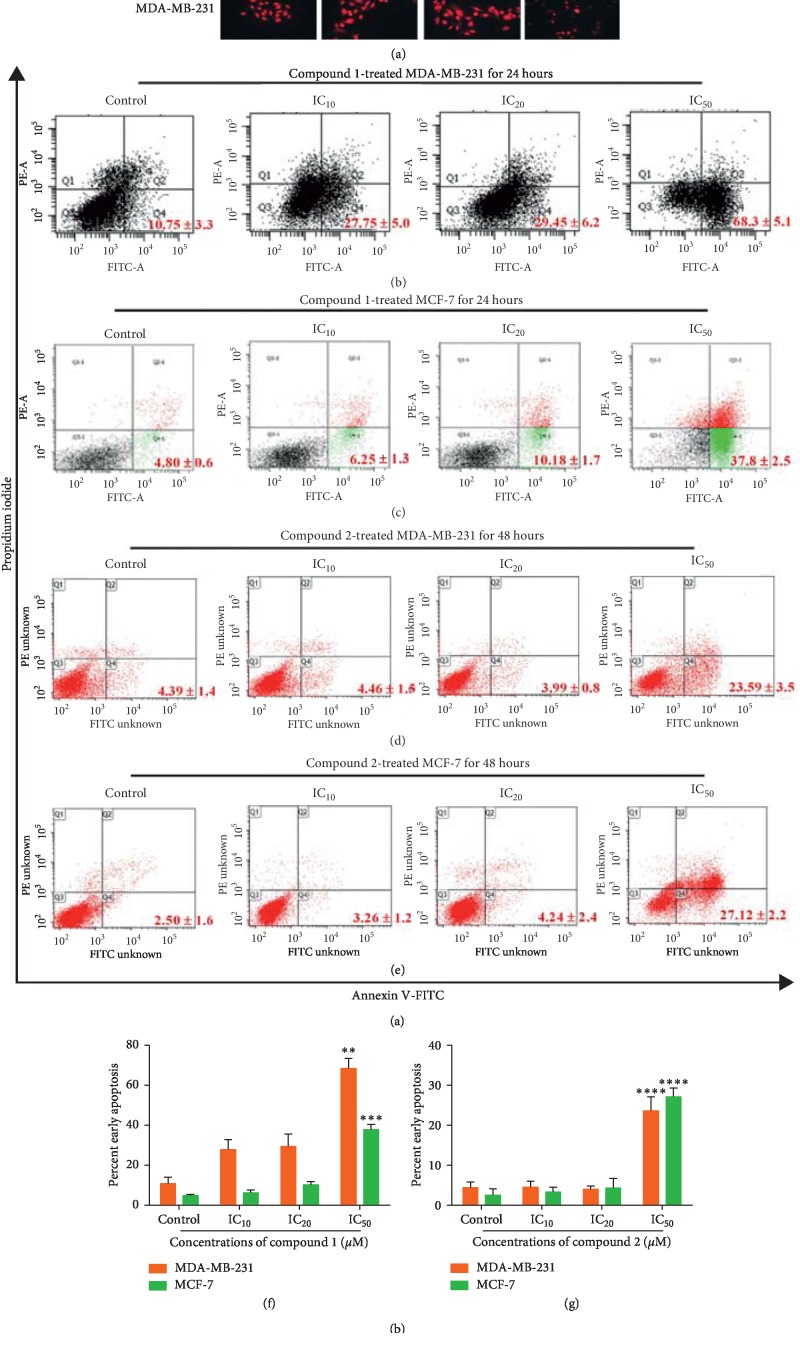
Apoptosis induced by dihydrochalcone derivatives. Cell morphology of apoptotic cells exhibited as condensed nuclei and apoptotic bodies (white arrows →) after compound 1 treatment for 24 hours and then staining with propidium iodide (PI) and examining under a fluorescence microscope (a). Apoptotic cells were treated with dihydrochalcone derivatives for 24 hours and then stained with annexin V-FITC and PI employing a flow cytometer as demonstrated by dot plots of MDA-MB-231 cells (b) and MCF-7 cells (c) and were treated with compound 2 for 48 hours in the MDA-MB-231 cells (d) and MCF-7 cells (e). Bar graphs of percent cells in early apoptosis of both types of cancer cells after compound 1 (f) and compound 2 (g) treatments are presented. ^*∗∗*^*p* < 0.01, ^*∗∗∗*^*p* < 0.001, and ^*∗∗∗∗*^*p* < 0.0001.

To examine the mode of cell death, during early apoptosis, the phosphatidylserine (PS) externalizes from the inner layer to the outer layer. In late apoptosis, the cell membrane loses its integrity and allows fluorescence dye to stain the DNA. By employing flow cytometer, the after-treatment of the cells with the reagent kit (Roche Diagnostics, Germany) included annexin V, a calcium-dependent phospholipid-binding protein with a high affinity for PS, and propidium iodide (PI) as a viable dye used to stain DNA, and to serve as an indicator of cell membrane integrity. PI binds to deoxyribonucleic acids after the cell membrane breaks down, revealing a difference between the early apoptotic cells (annexin V-positive and PI-negative) and the late apoptotic cells (annexin V-positive and PI-positive) [33]. MDA-MB-231 cells treated with compounds 1 and 2 showed the characteristics of apoptotic cells in terms of death morphology after treatment for 24 hours with nuclear condensation, fragmentation, and apoptotic bodies (white arrows in Figure 3(a)). Compound 1 and compound 2 also increased early apoptotic cell population. The percentage of early apoptotic cells is shown in the right lower quadrant and represents the early apoptotic cell population. This population increased in the MDA-MB-231 and MCF-7 cell lines after treatment with compound 1 for 24 hours (Figures 3(b) and 3(c)) and compound 2 at 48 hours (Figures 3(d) and 3(e)).

### 3.3. Reduction of Mitochondrial Transmembrane Potential (MTP) in Compound 1-Treated MDA-MB-231 and MCF-7 Cells

**Figure 4:**
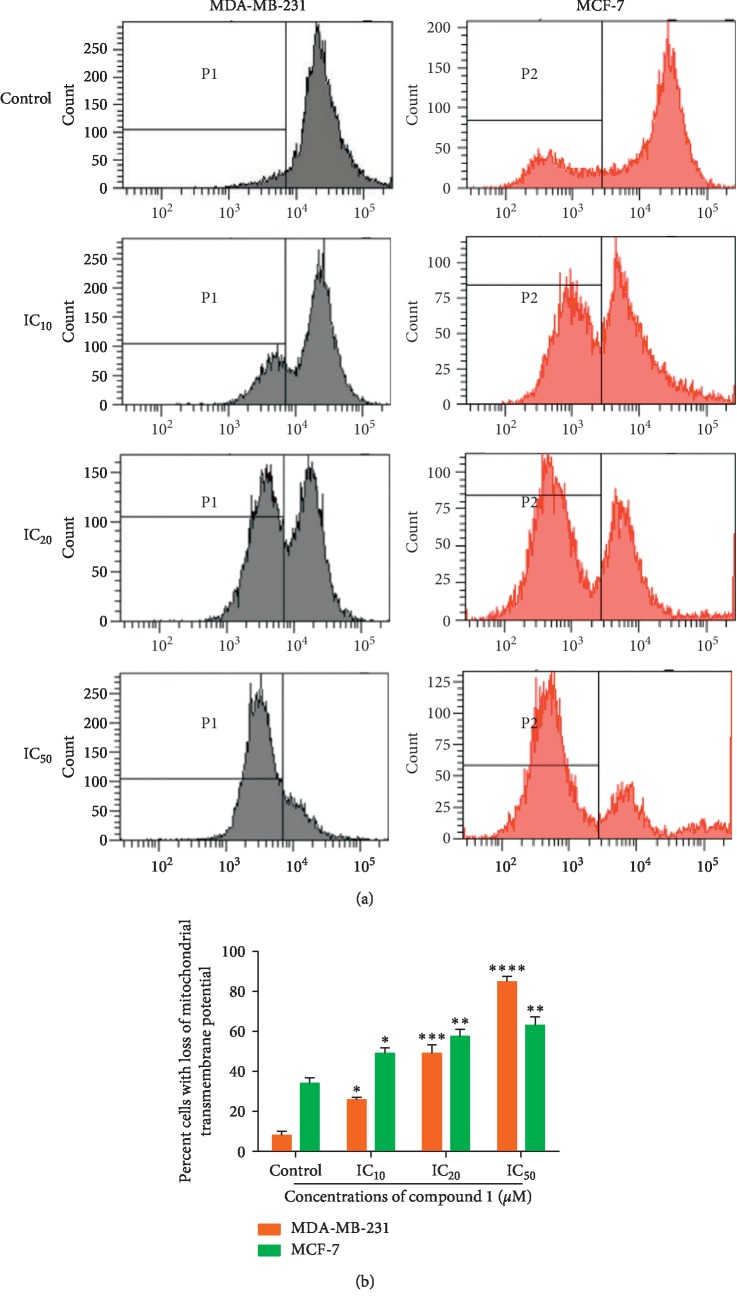
Reduction of mitochondrial transmembrane potential (MTP) of MDA-MB-231 and MCF-7 cells after treatment with compound 1. MDA-MB-231 and MCF-7 cells were treated with compound 1 at various concentrations for 24 hours, and MTP was measured by staining with DiOC_6_ and employing flow cytometry. The relative fluorescence intensity was measured. Histograms (a) and bar graphs (b) of percentage of both cancer cells with losses of MTP are presented. The statistical significance values compared to the control (without treatment) are marked with asterisks, ^*∗*^*p* < 0.05, ^*∗∗*^*p* < 0.01, ^*∗∗∗*^*p* < 0.001, and ^*∗∗∗∗*^*p* < 0.0001.

Compound 1 has been selected for further investigation of the mechanisms of cell death due to its high sensitivity toward both human breast cancer cells over compound 2 (calomelanone). In normal cells, the role of mitochondrial transmembrane potential (Δψm) is to maintain the function of the respiratory chain to generate ATP. During apoptosis, the mitochondrial transmembrane potential (Δψm) is disrupted, and the electrochemical gradient across the mitochondrial membrane then collapses. Several membrane permeable lipophilic cations, such as 3,3′-dihexyloxacarbocyanine iodide (DiOC_6_), have been used to determine the degree of mitochondrial transmembrane potential reduction [34]. After MDA-MB-231 and MCF-7 cells were treated with compound 1 for 24 hours, MTP was measured by staining cancer cells with DiOC_6_, which was taken up by the mitochondria and determined by a flow cytometer. The relative DiOC_6_ fluorescence level was high in normal cells but reduced in apoptotic cells. This occurred since the mitochondrial transmembrane permeability (MTP) is disrupted, and the dye leaks into the cytoplasm leading to lower fluorescence intensity. The percentage of cells experiencing a loss of MTP after compound 1 treatment for 24 hours is shown in Figure 4. The percentage of both MDA-MB-231 and MCF-7 cells with reduced MTP was significantly enhanced in a concentration-dependent manner.

### 3.4. Increased Cytosolic and Mitochondrial Ca^2+^ Levels in Compound 1-Treated MDA-MB-231 and MCF-7 Cells

**Figure 5:**
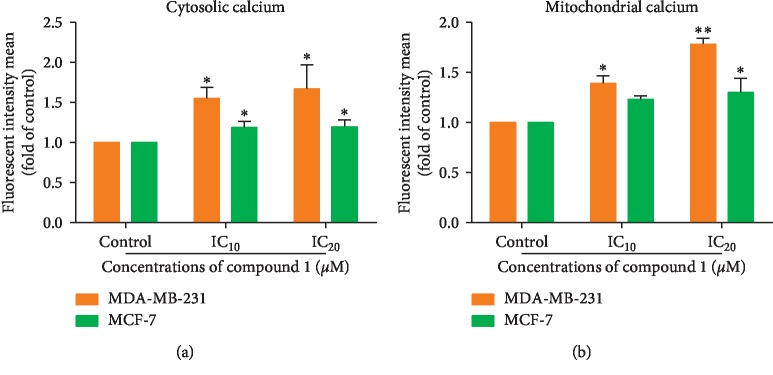
Alteration of cytosolic and mitochondrial Ca^2+^ levels in MDA-MB-231 and MCF-7 cells. After treatment with compound 1 for 24 hours, cancer cells were incubated with Fluo-3AM for 15 minutes to measure cytosolic Ca^2+^ (a). Mitochondrial Ca^2+^ was measured in MDA-MB-231 and MCF-7 cells after being treated cells with compound 1 for 24 hours and incubated with Rhod-2AM for 15 minutes (b). The cells were analyzed by employing flow cytometry. The data are shown as mean ± SD of fluorescence intensity as folds compared to the control (without treatment) from three independent experiments. The statistical significance value compared to the control (without treatment) is marked with asterisks, ^*∗*^*p* < 0.05 and ^*∗∗*^*p* < 0.01.

The apoptosis pathway is also mediated via endoplasmic reticulum (ER) stress with an increase of the cytosolic free Ca^2+^ level and in mitochondria [35–37]. Rhod-2AM with fluorescence excitation wavelength at 552 nm and emission wavelength at 581 nm is used to determine the Ca^2+^ level in mitochondria, whereas Fluo-3AM possesses an absorption spectrum compatible with excitation wavelength at 506 nm and emission wavelength at 526 nm that can be used to measure the intracellular cytosolic calcium level [38]. The Ca^2+^ level increased in compound 1-treated MDA-MB-231 and MCF-7 cells in both cytosolic (Figure 5(a)) and mitochondrial compartments (Figure 5(b)). Hence, compound 1 also induced human breast cancer cell apoptosis via the ER stress signaling pathway.

### 3.5. Induction of Caspase-3, Caspase-8 and, Caspase-9 Activities in Compound 1-Treated MDA-MB-231 and MCF-7 Cell Apoptosis

**Figure 6:**
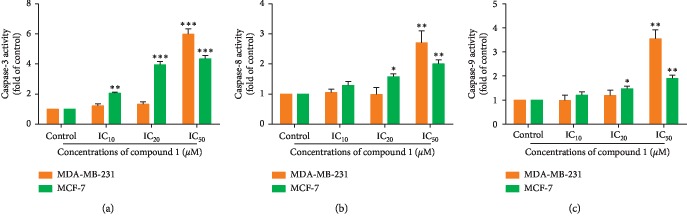
Caspase-3, caspase-8, and caspase-9 activities of MDA-MB-231 and MCF-7 cells after treatment with compound 1 for 24 hours. The caspase-3 (a), caspase-8 (b), and caspase-9 (c) activities were measured by using individual specific tetrapeptide substrates tagged with para-nitroaniline, and the absorbance of cleaved *p*-NA was determined by a spectrophotometric microplate reader. The statistical significance value compared to the control (without treatment) is marked with asterisks, ^*∗*^*p* < 0.05, ^*∗∗*^*p* < 0.01, and ^*∗∗∗*^*p* < 0.001.

The absorbance of chromophore *p*-nitroaniline (*p*-NA) was determined after cleavage from the labeled substrates DEVD-*p-*NA, IETD-*p-*NA, and LEHD-*p-*NA (substrates of caspase-3, caspase-8, and caspase-9, respectively). (Caspase-3/CPP32, Caspase-8/FLICE, Caspase-9/Mch6/Apaf-3 Colorimetric Protease Assays Kits); protocols from Invitrogen™, Camarillo California, were used to determine caspase activity using a spectrophotometric microplate reader. The *p*-NA absorbance was demonstrated with the yellow color absorbance spectrum at 405 nm. MDA-MB-231 and MCF-7 cells were treated with compound 1 at various concentrations for 24 hours, and the cells were then investigated for caspase activities. The caspase-3, caspase-8, and caspase-9 activities significantly increased in compound 1-treated MDA-MB-231 cells at a concentration of IC_50_. In the compound 1-treated MCF-7 cells, the caspase-3, caspase-8, and caspase-9 activities increased significantly in a dose-dependent manner (Figures 6(a)–6(c)).

### 3.6. Increased Levels of Proapoptotic Bcl-2 Family Proteins and Gene Expressions Involved in Cell Cycle Arrest

**Figure 7:**
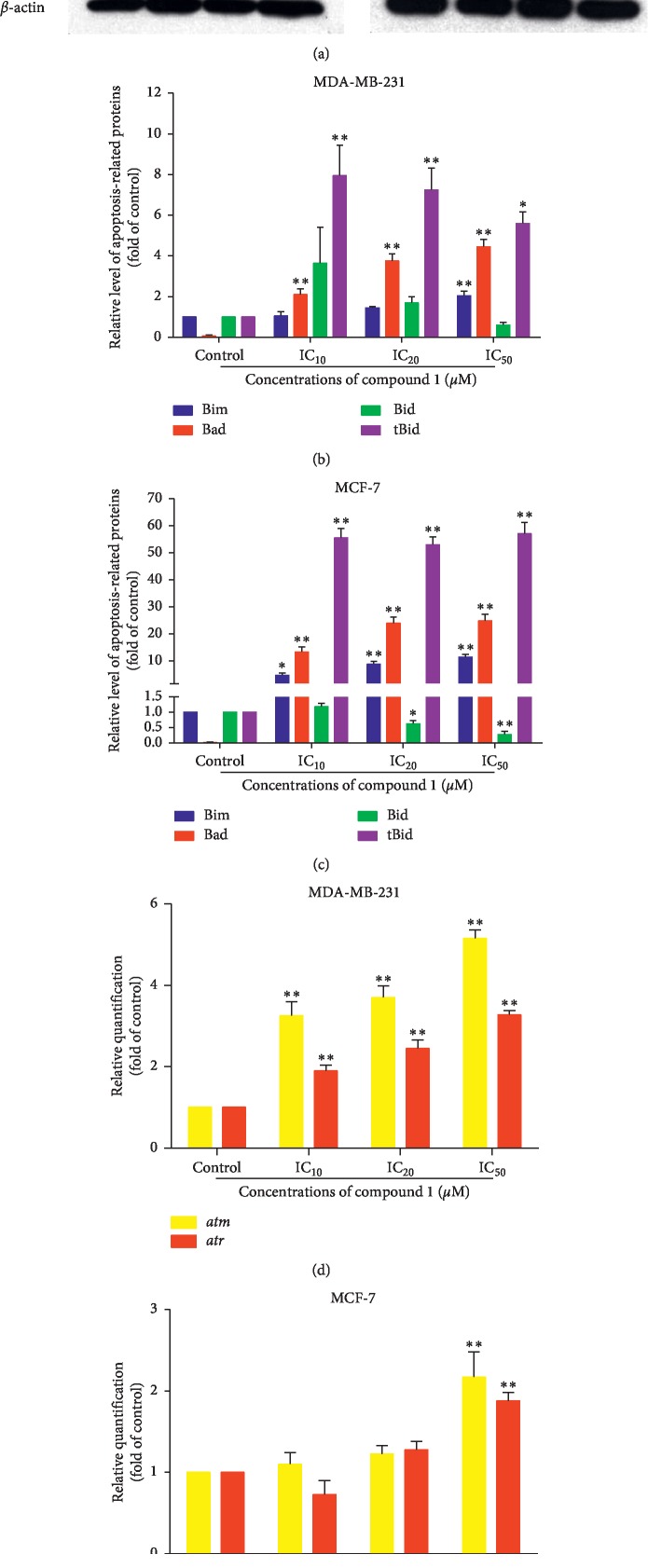
Alteration of apoptotic Bcl-2 family proteins and cell cycle checkpoint mRNA expressions in compound 1-treated MDA-MB-231 and MCF-7 cells. MDA-MB-231 and MCF-7 cells were treated with compound 1 at various concentrations for 12 hours. The expression levels of BH3-only proteins Bim, Bad, and Bid together with the truncated form (tBid) were demonstrated by Western blotting (a). The bands were folds when compared with the control as verified by using *β*-actin as a constitutive protein. The bar graphs of the relative levels of protein expressions of treated MDA-MB-231 cells (b) and MCF-7 cells (c) were obtained using densitometry from three independent experiments with the same results. Bar graphs are representative of *atm* and *atr* gene expressions in compound 1-treated MDA-MB-231 cells (d) and MCF-7 cells (e). The statistical significance values compared to the control (without treatment) are marked with asterisks, ^*∗*^*p* < 0.05 and ^*∗∗*^*p* < 0.01.

Determination of the expression of proapoptotic Bcl-2 family proteins including Bim, Bad, and tBid and gene expressions that were involved in cell cycle arrest was investigated after compound 1 treatment. Proapoptotic Bcl-2 family proteins were detected by Western blotting. The proapoptotic Bcl-2 family, Bim, Bad, and tBid significantly increased in compound 1-treated MDA-MB-231 and MCF-7 cells (Figures 7(a)–7(c)). The gene expressions that involve in cell cycle arrest, viz., *atm* and *atr* genes, were investigated by real-time reverse transcription-polymerase chain reaction (real-time RT-PCR). Gene expression levels of *atm* and *atr* significantly increased in compound 1-treated MDA-MB-231 cells at 12 hours in a dose-dependent manner (Figure 7(d)), whereas *atm* and *atr* gene expression levels were enhanced at IC_50_ after compound 1 treatment in MCF-7 cells (Figure 7(e)). ATM and ATR proteins are key regulators of the DNA damage response resulting in cell cycle arrest and cellular induction to apoptosis [39].

### 3.7. Compound 1 Induced Breast Cancer Cell Death via EGFR/MAPK Inhibition

**Figure 8:**
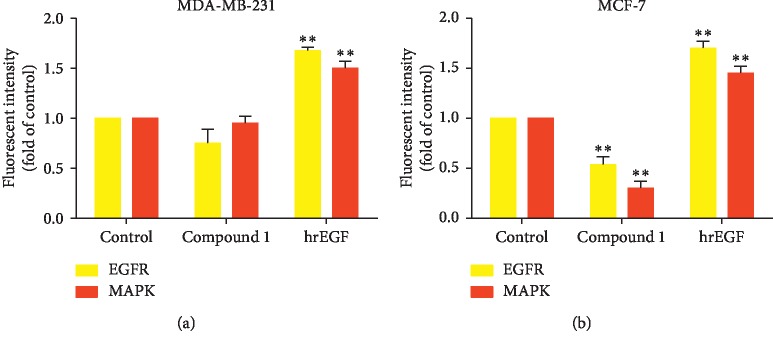
Inhibition of the EGFR/MAPK pathway after compound 1 treatment in MDA-MB-231 and MCF-7 cells. MDA-MB-231 and MCF-7 cells were incubated with compound 1 at IC_0_ and IC_50_ for 4 hours, and human recombinant EGF (hrEGF) was used as a positive control. Bar graphs of fluorescence intensity of EGFR and MAPK in MDA-MB-231 cells (a) and MCF-7 cells (b) were compared with the control (as folds) shown by using Guava® Flow Cytometry easyCyte^™^ Systems. The statistical significance values compared to the control (without treatment) are marked with asterisks, ^*∗∗*^*p* < 0.01.

To examine whether or not compound 1 induced apoptotic cell death via the EGFR/MAPK pathway, the EGFR/MAPK pathway was investigated using a FlowCellect™ EGFR/MAPK Activation Detection kit. After treating MDA‐MB‐231 and MCF‐7 with IC_50_ of compound 1 and comparing with positive control human recombinant EGF (hrEGF), it was found that the EGFR/MAPK signaling pathway was significantly diminished in MCF‐7 cells (Figure 8(a)). The EGFR/MAPK signaling pathway was significantly diminished in MCF-7 cells (Figure 8(b)), but was not altered in the MDA-MB-231 cells (Figure 8(a)).

### 3.8. Compound 1 and Chemotherapeutic Drug Combination Effect on MDA-MB-231 and MCF-7 Cells

**Figure 9:**
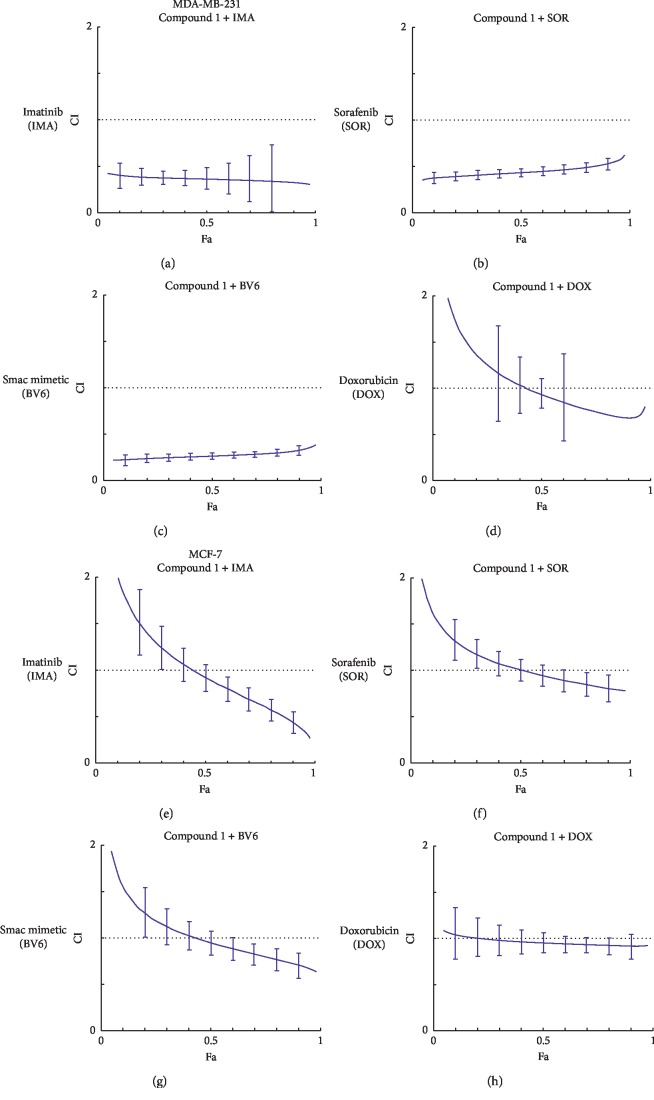
Combination indices of compound 1 and 4 chemotherapeutic drugs on MDA-MB-231 and MCF-7 cells. Compound 1-treated MDA-MB-231 cells were combined with imatinib (IMA) (a), sorafenib (SOR) (b), Smac mimetic (BV6) (c), and doxorubicin (DOX) (d), and compound 1-treated MCF-7 cells were combined with imatinib (IMA) (e), sorafenib (SOR) (f), Smac mimetic (BV6) (g), and doxorubicin (DOX) (h). Data were analyzed from CompuSyn Software analysis.

To determine the combined effects by computerized software between compound 1 and the chemotherapeutic drugs such as imatinib, sorafenib, Smac mimetic BV6, and doxorubicin on human breast cancer cells, it was found that in the MDA-MB-231 cells at Fa (fraction affected) = 0.5, combination indices (CI) were less than 1, which represented a synergistic effect except when compound 1 was combined with DOX, which produced an additive effect. However, at Fa = 0.9, in the MDA-MB-231 cells, all combination indices were less than 1, which was recognized as a synergistic effect (Figures 9(a)–9(d)). However, in MCF-7, at Fa = 0.5, all combination indices of compound 1 toward IMA, DOX, SOR, and BV6 were about 1, which were identified as an additive effect. In MCF-7 cells, at Fa = 0.9, all CI results were found to represent a synergistic effect, with the exception of a combination of compound 1 with DOX, which was identified as an additive effect (CI about 1) (Figures 9(e)–9(h)).

## 4. Discussion

Recently, there has been a report on a polyphenolic dihydrochalcone C-glucoside, a dihydrochalcone derivative that reduces the cardiotoxicity of doxorubicin in combined treatment through autophagy and effectively decreases the expression of p53/mTOR/p62 pathways [40]. The artificial sweetener, a neohesperidin dihydrochalcone, demonstrates anti-inflammatory and antiapoptosis effects against paraquat-induced liver injury in mice [41]. Two derivative dihydrochalcones, compound 1, 4′,6′-dihydroxy-2′,4-dimethoxy-5′-(2″-hydroxybenzyl)dihydrochalcone, and compound 2 (or calomelanone, 4′,6′-dihydroxy-2′,4-dimethoxydihydrochalcone), obtained from *Cyathostemma argenteum* possess anti-inflammatory activities [23]. Additionally, dihydrochalcone derivatives also contain antioxidant activity [42].

In the present study, two dihydrochalcone derivatives were compared for the inhibitory effect on cell growth/proliferation and mode/mechanism(s) of cell death. Compound 1 and calomelanone (compound 2) are derivatives of dihydrochalcone as shown in Figures 1(a) and 1(b). Compound 1 and 2 were toxic to both MDA-MB-231 and MCF-7 cells. These two compounds inhibited human breast cancer cell proliferation, viz., MCF-7 and MDA-MB-231 cells. They were found to be less toxic to normal peripheral blood mononuclear cells (PBMCs) and murine normal fibroblast NIH3T3 cells. The results of the MTT assay indicated the sensitivity of the compound in various cells as follows: MCF-7 > MDA-MB-231 > NIH3T3 > PBMC cells. Compound 1 was found to be more toxic than compound 2 due to less IC_50_ value when compared to each breast cancer cell line at 24 hours; however, compound 2 significantly reduced IC_50_ concentrations in both cells at 48 hours when compared to those in the 24-hour treatment; hence, the toxicity was determined to be time-dependent.

The morphological changes of the treated cells were altered as nuclear condensation, fragmentation, and apoptotic bodies (white arrows in Figure 3(a)) when stained with propidium iodide (PI) and examined under a fluorescence microscope. To confirm dihydrochalcone-induced cell apoptosis in both breast cancer cells, the cells were stained with annexin V-FITC/PI employing the flow cytometry technique. It was determined that the mode of MDA-MB-231 and MCF-7 cell deaths that were induced by both dihydrochalcones at 24 hours (Figures 3(b) and 3(c)) and in compound 2 at 48 hours (Figures 3(d) and 3(e)) provided the same results of apoptosis. The percentage of the right lower quadrant, which represents the early apoptotic cell population, significantly increased in both MDA-MB-231 and MCF-7 cells at IC_50_ after compound 1 and 2 treatments as shown in Figures 3(f) and 3(g).

Apoptosis, a regulated and programmed form of cell death, involves multiple factors. Caspases play a major and central role in apoptotic mechanisms [43]. In apoptosis via the intrinsic pathway, cells respond to multiple intracellular stress factors, such as chemotherapeutic drugs, radiation, free radicals, and toxins, allowing the alteration of the mitochondrial outer membrane permeabilization (MOMP) [44] to be disrupted and leading to a decrease in mitochondrial transmembrane potential (MTP or Δψm). Compound 1 induced the reduction of MTP, indicating mitochondrial pathway-mediated apoptosis in both MDA-MB-231 and MCF-7 cells (Figure 4). The percentage of cells with reduced MTP increased in a dose-dependent manner in both cells.

The role of calcium ion (Ca^2+^) is the ubiquitous second messenger that controls a broad variety of physiological events. Fine regulation of intracellular Ca^2+^ homeostasis by anti- and proapoptotic proteins, such as the Bcl-2 family proteins, alters the signaling to which mitochondria and other organelles or cellular effectors are exposed and therefore affects various modes of cell death induction [37, 45]. The ER stress pathway exists via an increase in Ca^2+^ levels in the mitochondrial and/or cytosolic compartments. Compound 1 induced the enhancement of Ca^2+^ levels in the cytosol (Figure 5(a)) and mitochondria (Figure 5(b)) in both the MDA-MB-231 and MCF-7 cells in a dose-dependent manner. Rhod-2AM is used to determine the presence of calcium ions in the mitochondria, whereas Fluo-3AM is applied to detect the intracellular cytosolic calcium levels. This indicates that the mechanism of apoptotic cell death of human breast cancer MDA-MB-231 and MCF-7 cells was also conducted via the ER stress pathway.

When initiator caspases are activated, it will activate the downstream effector caspases by proteolysis [46]. The effector caspases (caspase-3, caspase-6, and caspase-7) then cleave their substrates, such as poly(ADP-ribose)polymerase (a DNA repairing enzyme), actin, lamin, and fodrin, resulting in changes of cellular morphology and the biochemical characteristics of apoptosis [47]. The initiator caspase-8 and caspase-10 are involved in the extrinsic or death receptor pathway, whereas caspase-9 is related to the intrinsic or mitochondrial pathway. The effector caspase-3 is activated as an executioner or effector caspase in the final common pathway [48, 49]. After treatment with compound 1 in MDA-MB-231 and MCF-7 cells for 24 hours, caspase-3, caspase-8, and caspase-9 activities significantly increased in the MDA-MB-231 cells, especially at the concentration of IC_50_. However, compound 1-treated MCF-7 cells induced caspase-3, caspase-8, and caspase-9 activities significantly in a dose-dependent manner (Figures 6(a)–6(c)). Taken together, this indicates that MDA-MB-231 and MCF-7 cells were induced to undergo apoptosis via both the intrinsic and extrinsic pathways.

Apoptosis is initiated within the cells and regulated by a group of proteins that belong to the Bcl-2 family. There are three groups of Bcl-2 family proteins (proapoptotic multidomain, proapoptotic BH3-only, and antiapoptotic proteins) that influence the apoptotic pathway. Proapoptotic multidomain proteins, e.g., Bax and Bak, and proapoptotic BH3-only proteins, such as Bim, Bad, Bid, and tBid, promote apoptosis; whereas antiapoptotic proteins, namely, Bcl-2 and Bcl-xL, block apoptotic cell death. The initiation of apoptosis depends on the balance between the pro- and antiapoptotic proteins [50, 51]. This is referred to as rheostat, which involves switching on the apoptosis when antiapoptotic protein levels are less than the proapoptotic proteins. Binding of the activator BH3-only proteins (such as Bid and Bim) to mitochondrial membranes increases their affinity for the pore formers (such as Bax and Bak), which are activated and lead to the permeabilization of the mitochondrial outer membrane to decrease mitochondrial transmembrane potential (MTP) and to allow for apoptotic protein release. The sensitizers, BH3-only proteins, such as Bad, Bid, and Bim, bind and inhibit antiapoptotic proteins (Bcl-2) resulting in cellular apoptosis [52, 53]. From these results, it was determined that the proapoptotic BH3-only proteins, viz., Bim, Bad, and truncated Bid (tBid), were significantly increased in the compound 1-treated MDA-MB-231 and MCF-7 cells (Figures 7(a)–7(c)). Bid bridges the cross-talk between the extrinsic and intrinsic pathways through its cleavage by caspase-8 to become tBid [54]. The results of immunoblotting showed that the Bid protein level decreased, whereas tBid increased. This confirmed that compound 1 induced breast cancer cells apoptosis via the intrinsic and extrinsic pathways through the expression of Bim, Bad, and tBid proapoptotic proteins and then accelerated the pore formation at the outer mitochondrial membrane and activated initiator caspase-9 and executioner caspase-3 as the sequelae for apoptosis induction.

ATM (ataxia telangiectasia mutated) is a transmembrane serine/threonine kinase and ATR (ataxia telangiectasia and Rad3 related) signaling is affected by the tumorigenesis. ATM and ATR protect cells that are obtained from tumor progression by inducing cell cycle arrest and apoptosis in the early phases of tumorigenesis [55]. In precancerous lesions, the ATM and ATR pathways are activated to help the cells resist the progression of tumors [56]. Moreover, in the loss of ATM or ATR functions, genome instability such as mutation or deletion will promote cell survival, potentially resulting in cancerous formations and ultimately tumor promotion [39]. ATM is recruited and activated by DNA double-strand breaks, and ATR is activated in response to single-strand breaks. ATM and ATR phosphorylate several key proteins, which initiate the activation of p53 in response to DNA damage to promote cell cycle arrest [57]. After compound 1 treatment for 12 hours, *atm* and *atr* mRNA expressions were measured by real-time RT-PCR. The expression levels of *atm* and *atr* mRNAs were significantly increased in MDA-MB-231 cells in a dose-dependent manner (Figure 7(d)), but in MCF-7 cells, both genes significantly increased only at a concentration of IC_50_. This suggests that compound 1 induced cell apoptosis via cell cycle arrest by increasing *atm* and *atr* mRNA expressions.

The MAPK pathway is one of the most important regulatory mechanisms in eukaryotic cells. After activation by upstream kinases, different subfamilies regulate various physiological processes in the cells, including inflammation, stress, cell growth, development, cell differentiation, and death, through multiple substrates, for example, phosphorylated transcription factors [58]. Recently, several studies provide new insights on p38, JNK, and MAPK pathway functions in control of the homeostasis of autophagy and apoptosis in response to genotoxic stress [59]. The epidermal growth factor receptor (EGFR) is one of the receptor tyrosine kinases that can activate the MAPK pathway [60]. Growth factors and mitogens use the Ras/Raf/MEK/ERK signaling cascade to transmit signals from their receptors to regulate gene expression and prevent apoptosis [61]. EGFR/MAPK activity was measured after compound 1 treatment for 4 hours, and human recombinant EGF (hrEGF) was used as a positive control by using FlowCellect™ EGFR/MAPK Activation Detection kit employing Guava® Flow Cytometry easyCyte™ Systems. The results revealed that the degree of EGFR/MAPK activation was significantly decreased in MCF-7 cells (Figure 8(b)), but did not change in the MDA-MB-231 cells (Figure 8(a)). Potentially, there is a high expression of EGFR in MDA-MB-231 cells, which causes MDA-MB-231 to be invasive to cancer cells. The EGFR/MAPK pathway targets the inhibition of cell proliferation and induces cell apoptosis; hence, compound 1 was efficient in inducing apoptotic cell death via the inhibition of the EGFR/MAPK pathway.

This study is the first to report that compounds 1 and 2 could induce human breast cancer MDA-MB-231 (invasive) and MCF-7 (noninvasive) cytotoxicity via apoptotic cell death. Notably, compound 1 was found to be more toxic than compound 2, as was evaluated from 50% of inhibitory concentration at 24 hours, and this was compared to that of compound 2 at 48 hours. The molecular mechanisms of cell death involved in extrinsic, intrinsic, ER stress, caspase-9, caspase-8, and caspase-3 induced *atm* and *atr* gene expressions and Bcl-2 family protein expression, whereas the EGFR/MAPK pathway was ameliorated in noninvasive breast cancer MCF-7 but not in invasive MDA-MB-231 cells. Therefore, compound 1 obtained from *Cyathostemma argenteum* is a potentially active compound that can be used for anticancer agent development with less cytotoxicity upon normal cells. The difference in the chemical structure between compounds 1 and 2 is the side chain of the hydroxybenzyl group that is included in compound 1, but not in compound 2. Furthermore, the dihydroxy and dimethoxy groups are also present in different positions in both active compounds when compared, which demonstrates the structure-activity relationship (SAR).

## 5. Conclusion

**Figure 10:**
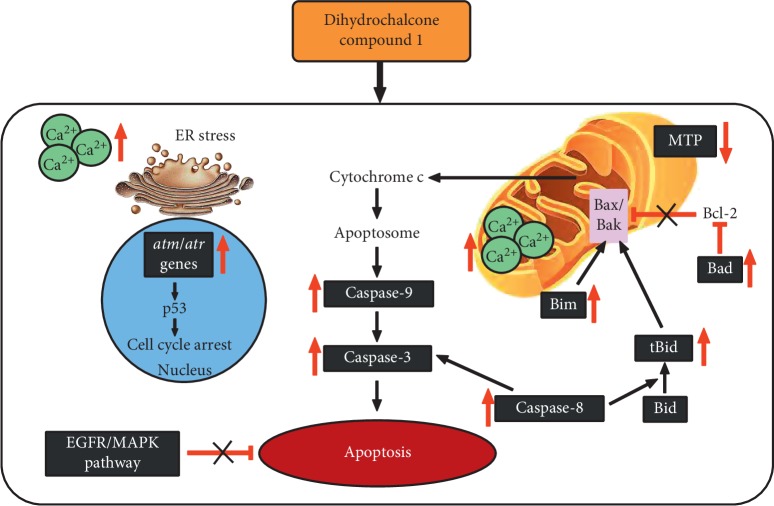
Illustration representing the mechanisms of compound 1-induced human breast cancer cell apoptosis.

Compound 1, a dihydrochalcone derivative, is an anticancer agent that induced MDA-MB-231 and MCF-7 cell apoptosis via the mitochondrial pathway by reducing mitochondrial transmembrane potential, increasing caspase-9 activity, and increasing BH3-only proapoptotic proteins such as Bim, Bad, and tBid level. The extrinsic pathway was also induced via caspase-8 activity and the cleavage of Bid. The function of the ER stress pathway was evidenced by increasing calcium ion levels in both the mitochondria and cytosolic compartments. Compound 1 also induced cell cycle arrest via increased *atm* and *atr* checkpoint gene expression levels and inhibited the EGFR/MAPK survival pathway to promote cell apoptosis (Figure 10).

## Figures and Tables

**Table 1:** Primers for specific genes in the real-time RT-PCR method.

Symbol	Synonym	Name		Sequence of primer (5′⟶3′)
*atr*		ATR serine/threonine kinase	F	GGGATGCCACTGCTTGTTATGAC
			R	CTGTCCACTCGGACCTGTTAGC
*atm*		ATM serine/threonine kinase	F	TGCCAGACAGCCGTGACTTAC
			R	ACCTCCACCTGCTCATACACAAG
*gapdh*		Glyceraldehyde-3-phosphate dehydrogenase	F	TGCACCACCAACTGCTTAGC
			R	GGCATGGACTGTGGTCATGAG

**Table 2:** Inhibitory concentrations of compound 1 at 24 hours.

Cell types	Concentrations of compound 1 (*μ*M)		
	IC_10_	IC_20_	IC_50_
MDA-MB-231	57.1 ± 3.5	84.8 ± 2.8	232.7 ± 3.9^*∗∗∗∗*^
MCF-7	22.8 ± 3.7	45.7 ± 4.8	88.3 ± 5.4
NIH3T3	—	—	>200
PBMCs	—	—	>200

^*∗∗∗∗*^ *p* < 0.0001, IC_50_ of compound 1-treated MDA-MB-231 compared with compound 1-treated MCF-7 at 24 hours.

**Table 3:** Inhibitory concentrations of compound 2 at 24 and 48 hours.

Cell types	Concentrations of compound 2 (*μ*M)					
	24 hours			48 hours		
	IC_10_	IC_20_	IC_50_	IC_10_	IC_20_	IC_50_
MDA-MB-231	—	—	>300	14.0 ± 2.1	31.1 ± 3.4	85.9 ± 5.3^*∗∗∗∗*^
MCF-7	25.2 ± 5.6	42.9 ± 3.3	75.7 ± 0.2^*∗∗∗*^	15.2 ± 2.5	22.8 ± 2.5	46.2 ± 4.7

^*∗∗∗∗*^ *p* < 0.0001, IC_50_ of compound 2-treated MDA-MB-231 compared with that of compound 2-treated MCF-7 at 48 hours. ^*∗∗∗*^*p* < 0.001, IC_50_ of compound 2-treated MCF-7 at 24 hours compared with that of compound 2-treated MCF-7 at 48 hours.

## Data Availability

The data collected in the present study are properly analyzed and summarized in Methods and Results, and all are available from the corresponding author upon reasonable request. All materials used in this study are properly included in Methods.
